# Physiological consequences of doublet discharges on motoneuronal firing and motor unit force

**DOI:** 10.3389/fncel.2015.00081

**Published:** 2015-03-10

**Authors:** Włodzimierz Mrówczyński, Jan Celichowski, Rositsa Raikova, Piotr Krutki

**Affiliations:** ^1^Department of Neurobiology, University School of Physical EducationPoznań, Poland; ^2^Institute of Biophysics and Biomedical Engineering, Bulgarian Academy of SciencesSofia, Bulgaria

**Keywords:** doublet, motoneuron, interspike interval, motor unit, force development

## Abstract

The double discharges are observed at the onset of contractions of mammalian motor units (MUs), especially during their recruitment to strong or fast movements. Doublets lead to MU force increase and improve ability of muscles to maintain high force during prolonged contractions. In this review we discuss an ability to produce doublets by fast and slow motoneurons (MNs), their influence on the course of action potential afterhyperpolarization (AHP) as well as its role in modulation of the initial stage of the firing pattern of MNs. In conclusion, a generation of doublets is an important strategy of motor control, responsible for fitting the motoneuronal firing rate to the optimal for MUs at the start of their contraction, necessary for increment of muscle force.

## Introduction

A pair of action potentials at short interspike intervals (ISIs, below 10 ms) called “doublet” (Simpson, 1969) has been frequently observed at the beginning of discharge pattern of motoneurons (MNs). Such initial doublets in trains of action potentials of motor units (MUs) have been recorded from numerous human muscles during different types of voluntary activity (Person and Kudina, 1972; Kudina, 1974; Bawa and Calancie, 1983; Kudina and Alexeeva, 1992; Garland and Griffin, 1999) or from animal muscles during locomotion (Zajac and Young, 1980b; Hennig and Lømo, 1985; Hoffer et al., 1987; Gorassini et al., 2000). Existence of doublets has also been confirmed in electrophysiological studies performed on MNs innervating inspiratory (Kirkwood and Munson, 1996) and locomotor muscles (Spielmann et al., 1993) of cat or hind limb muscles of rat (Mrówczyński et al., 2010; Bączyk et al., 2013) during their activation with intracellular current injection.

Many experiments on human muscles (Bawa and Calancie, 1983; Kirkwood and Munson, 1996; Van Cutsem et al., 1998; Garland and Griffin, 1999; Christie and Kamen, 2006) and MUs of various animal species (Zajac and Young, 1980a; Hennig and Lømo, 1987; Sandercock and Heckman, 1997) have suggested that doublets are responsible for considerable enhancement of muscle output force. From this reason, doublets are considered as a special strategy of the central nervous system, which improves efficiency of a motor task requiring large force especially at early stage of muscle contraction (Garland and Griffin, 1999; Kudina and Andreeva, 2013).

The occurrence of doublets is also an important mechanism of adaptation to increasing level of muscle activity. Binder-Macleod and Barker (1991) have demonstrated that effects of doublet in force enhancement are greater in fatigued than in unfatigued muscles. Furthermore, a substantial increase of a number of doublets in muscles of trained athletes during dynamic voluntary contractions have suggested their contribution to an increase in the speed of contraction after the dynamic training (Griffin et al., 1998; Van Cutsem et al., 1998).

The paper aims to describe physiological consequences of the doublet occurrence for the afterhyperpolarization (AHP) parameters following the action potentials and consequently for initial ISIs in a pattern of motoneuronal discharges, which have not been described in previous reviews concerning the doublets (Garland and Griffin, 1999; Kudina and Andreeva, 2013). The consequences of the doublet discharges are discussed in relation to the MU force development.

## The Incidence of Doublets in Motoneurones

Experimental data obtained in animal studies suggest that the ability to produce doublets is attributed rather to fast than to slow MNs (Gorassini et al., 2000). However, electrophysiological studies with the intracellular injection of depolarizing current into spinal MNs of cat (Spielmann et al., 1993) and rat (Mrówczyński et al., 2010; Bączyk et al., 2013) have shown that doublets were produced by both slow and fast MNs. Therefore, it is likely that specific organization of supraspinal pathways descending rather to fast than slow MNs is responsible for doublet discharges during strong contractions in natural conditions.

Some studies indicate that the occurrence of doublets in the pattern of motoneuronal discharges depends on motoneuronal excitability (Christie and Kamen, 2006) and on power of synaptic inputs to MNs (Gorassini et al., 2000). Experiments with intracellular injection of a depolarizing current to rat MNs have demonstrated that doublet discharges are generated at current intensity 2.1–2.4 and 2.1–3.24 times higher than the rheobase of fast and slow MNs, respectively (Mrówczyński et al., 2010; Bączyk et al., 2013). In electrophysiological experiments, the depolarizing current is considered as a physical equivalent of the total synaptic input (Baldissera et al., 1987; Binder and Powers, 1999). From this point of view, a rapid increase of postsynaptic activity evoked through descending drive in MNs seems to be a major factor enabling generation of doublets during strong movements.

## Changes in the Firing Pattern and the After Hyperpolarization After the Doublet Discharge

The neuronal firing rate is regulated by several mechanisms. The spike frequency adaptation (SFA) is one of fundamental neuronal properties influencing their repetitive firing. It indicates a decrease in action potentials discharge rate over time (Miles et al., 2005). A period including the first few spikes of a motoneuronal firing has been determined as an “initial” phase of SFA and it is followed by an “early” (up to 250 ms) and “late” (from seconds to even minutes) adaptations (Granit et al., 1963; Kernell and Monster, 1982; Sawczuk et al., 1995; Powers et al., 1999). The initial high rate of motoneuronal firing is responsible for the increase of speed of force development at the onset of a MU contraction, and despite a decreased firing rate observed during the early and late adaptation phases MUs are still able maintain relatively steady level of force (Burke et al., 1976; Stein and Parmiggiani, 1979; Bigland-Ritchie et al., 1983).

**Figure 1:**
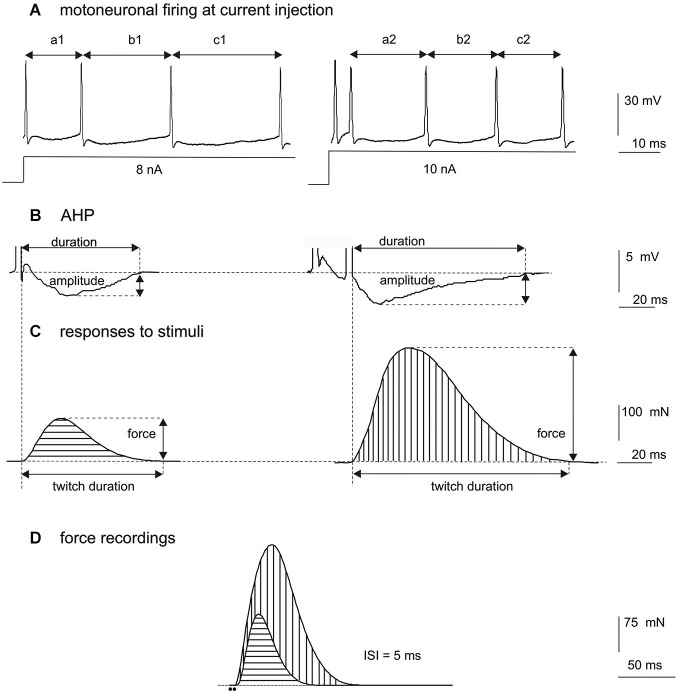
**(A)** Comparison of discharge rates of a single hind-limb MN of rat under pentobarbital anesthesia, after intracellular injection of depolarizing current of 8 nA (left record, without a doublet) and 10 nA (right record, with a doublet at about 5 ms ISI). Note the increased firing frequency of the MN with increased depolarizing current (the mean of a1 + b1 + c1 is longer than the mean of a2 + b2 + c2), however, the ISI immediately following the doublet (a2) is longer than the ISI after a single pulse below the doublet threshold (a1), and is longer than mean ISI calculated for later discharges (b2 + c2). **(B)** Comparison of the afterhyperpolarization (AHP) amplitude and duration after a single pulse (left record) or a doublet of action potentials (right record), showing a prolongation of the AHP and an increase of the AHP amplitude following a doublet at 5 ms ISI. **(C)** Models of twitch-shape contractions mathematically subtracted from the contraction obtained by two consecutive pulses at 5 ms ISI, for a fast-type MU. On the left, the twitch record in response to a single stimulus, on the right the response to the second stimulus calculated as a difference between the two superimposed recordings in **(D)**. Note higher force and longer duration of twitch-shape response to the second stimulus. The beginning of each record corresponds to the appearance of a stimulus delivered to the axon. **(D)** Superimposed MU force records (the same MU as in **C**) obtained by application of one pulse (horizontal hatching) or by two consecutive pulses delivered at 5 ms ISI (vertical hatching). Note evidently increased MU force after the doublet. The time position of two stimuli at 5 ms ISI is indicated by dots below the record.

The firing rate depends also on the excitation intensity. Studies with the intracellular injection of depolarization current into cat (Spielmann et al., 1993) or rat motoneurones (Mrówczyński et al., 2010) have demonstrated an increase of the overall firing frequency of a MN with increasing intensity of applied current (Figure 1A). However, after doublet, a prolongation of the following ISI is observed, causing a transient decrease of firing rate (compare a2 vs. a1 in Figure 1A). This reduction of the firing rate of MNs appears despite higher intensity of depolarization current applied. After all, direct comparison of discharge patterns without doublets (evoked at a lower intensity of intracellular depolarizing current or a weaker synaptic input to MNs) to those with doublets (evoked at a higher intensity of depolarizing current or a stronger synaptic input to MNs) seems insufficient to explain functional consequences of initial doublets.

A considerable variability of ISIs in motoneuronal firing evoked by stretching of a muscle and activation of proprioceptors has been observed (Kostyukov et al., 2009, 2011). According to these reports, this is related to non-linear processes of summation of consecutive AHPs, described earlier by Baldissera and Gustafsson (1974).

Studies on rat MNs stimulated antidromically by one pulse and by doublet of pulses at intervals from 5 to 10 ms have shown that doublet modulates the AHP duration and amplitude in both types of MNs (Figure 1B; Mrówczyński et al., 2007). In MNs antidromically stimulated with trains of stimuli, from one to five, applied at 5 ms ISIs, an evident increase of the AHP amplitude and a significant prolongation of the AHP duration have been demonstrated after a doublet or sometimes also after a triplet of stimuli (Mrówczyński et al., 2011; Krutki et al., 2014). The AHP duration has not been considerably modified by the following (4th and 5th) pulses in the train. The results indicate that at high stimulation frequency the second activation has the strongest effect on the AHP course.

Duration of the AHP is an important factor influencing a rate of neuronal discharges (Eccles et al., 1958). A considerable decrease of motoneuronal excitability during the AHP reduces the probability of occurrence of subsequent action potential (Kernell, 1965). From this reason the AHP duration is extremely important property of MNs, which controls their firing rate (Piotrkiewicz et al., 2007) and in addition can be used to differentiate fast (with short AHP) and slow MNs (with long AHP) (Gardiner, 1993).

The AHP is an effect of increased potassium conductance in a neuron following the action potential (Barrett et al., 1980). Therefore, it is supposed that the post-doublet prolongation of the AHP duration is an effect of increase in the potassium conductance. However, many studies on spinal MNs and ascending neurons (Kuno et al., 1970; Baldissera and Gustafsson, 1974; Baldissera et al., 1978; Gustafsson, 1984; Mrówczyński et al., 2008) have demonstrated a non-linear summation of the AHPs after the doublet activation. These results have implied that additional ionic mechanisms are involved in prolongation of the AHP duration after the doublet than those responsible for the AHP following a single action potential. It is likely that activation of special types of potassium channels responding to increased intracellular concentration of sodium ions (K^Na+^ channels) may contribute to the increase of potassium conductance. Such channels have low sensitivity to normal cytoplasmatic concentration of sodium ions and are not involved in production of a single action potential. According to Safronov and Vogel (1996), a short train of stimuli delivered to the neuron can evoke an intracellular accumulation of sodium ions that is necessary to activate the K^Na+^ channels.

The increase of the AHP duration following the doublet seems to be responsible for a temporary reduction of motoneuronal firing rate, and therefore may be considered as important physiological mechanism fitting the motoneuronal firing rate to that optimal for MUs. Thus, this is an additional internal mechanism of motoneuronal firing rate reduction to previously described mechanisms related to the neuronal network activity, as reciprocal inhibition from the Renshaw cells, inhibition by Ib interneurons (from Golgi tendon organs) or inhibition from interneurons receiving information from descending pathways (Jankowska and Roberts, 1972; Hultborn et al., 1988; Jami, 1992).

## The Influence of a Doublet on MU Force Development

The doublet of stimuli at the beginning of a train of pulses leads to an increase of the force output of contracting MU. Such effects have been frequently observed in experiments with doublets of pulses (in a range of 5–10 ms) on isolated fast or slow MUs of hind limb muscles of cat (Burke et al., 1976; Stein and Parmiggiani, 1979; Zajac and Young, 1980a) and rat (Hennig and Lømo, 1987; Celichowski and Grottel, 1998). A potentiation of MUs force in response to doublet has resulted from non-linear summation of twitch forces (Duchateau and Hainaut, 1986a) and could be twice to three times higher than the force of a twitch evoked by a single pulse (Parmiggiani and Stein, 1981; Kamavuako and Farina, 2010).

Simultaneous recording of the action potential from a single MN and the twitch of muscle fibers innervated by that neuron has revealed a positive correlation between the AHP duration and the twitch duration, and this correlation has been documented in several species, as cat (Zengel et al., 1985; Cope et al., 1986), rat (Gardiner and Kernell, 1990) or mouse (Meehan et al., 2010). All these studies have revealed that the activation with a single stimulus results in longer AHP and longer twitch of slow MUs in relation to fast ones. However, the amplitude and duration of both the AHP (Gustafsson, 1984; Mrówczyński et al., 2011) and twitch-shape responses (obtained by a mathematical decomposition of the recorded tetanus) to successive activations (Celichowski et al., 2008) are not constant. Recently, Krutki et al. (2014) have demonstrated in rat a parallelism in modification of the AHP as well as the contraction time and amplitude of the twitch-shape responses to individual stimuli. Parameters collected in one series of experiments with intracellular recordings of MNs (AHP amplitude and duration) (Figure 1B) have been compared to data from another series of experiments with the MU force recordings (Figure 1D) and to results of their mathematical decomposition (amplitude and duration of twitch-like responses to individual stimuli) (Figure 1C). In both series of experiments MNs as well as MUs were activated by trains of stimuli with the increasing numbers of pulses, from one to five, delivered at 5 ms ISIs. The most noticeable changes (the increase in the amplitude and the duration) have been observed in both the AHP and twitch-shape response parameters as an effect of activation with two stimuli (Figures 1B,C).

According to Krutki et al. (2014) an increase of twitch force in MUs after the doublet results rather from intracellular processes within muscle fibers than from electromechanical-coupling mechanisms. Duchateau and Hainaut (1986b) have suggested that an intensification of membrane processes in muscle fibers, leading to an increase of calcium concentration in the cell cytosol, is a cause of post-doublet twitch potentiation. Recently, Cheng et al. (2013) have pointed out that doublets evoked an increase of the Ca^2+^ release from sarcoplasmic reticulum, which is accompanied by greater force production in unfatigued muscle fibers of mouse. Thus, the increase of Ca^2+^ release enabling the phosphorylation of myosin light chain is responsible for facilitated formation of additional force-bearing cross bridges in the vicinity of already attached cross bridges leading to increase of MUs twitch force following the doublet (Sweeney et al., 1993; Abbate et al., 2002).

## Functional Implications of Doublet

The initial doublet is a specific pattern of MN discharges described by Binder-Macleod (1995) as “high to low” strategy, with a transition from high to low discharge rate. Such patterns contain a strong initial dynamic component followed by the steady state activity. According to the hypothesis by Kostyukov and Korchak (1998), dynamic components in the efferent commands play a decisive role in coding the final position of limbs in real movements.

However, it should be stressed that during voluntary activity, a strong descending drive to MNs causes recruitment of many additional MUs (Aagaard, 2003). Some studies concerning human training have demonstrated that strong MUs may be included into the muscle contraction at an early stage of force development (Van Cutsem et al., 1998; Kamen and Knight, 2004; Vila-Chã et al., 2010). Therefore, although the recruitment is the main mechanism of force regulation, doublets add an extra force to the muscle contraction. However, Sandercock and Heckman (1997) have reported that muscle movement completely abolishes muscle potentiation evoked by doublet after about 0.4 s of eccentric or concentric contractions of cat soleus muscle. Such result suggests that the doublet can evoke an initial force increment, but this effect does not remain high throughout the movement (Garland and Griffin, 1999). Thus, the physiological meaning of doublets in the force increase could be less significant during voluntary activity than it appears from the traditional scheme based on comparison of linear summation of two isolated isometric twitches. Moreover, doublets are not unique components responsible for adding force at the beginning of MUs contraction. Gorassini et al. (2000) have noticed a variety of high-frequency firing patterns started with triplets in fast MUs during locomotion of rats. Thus, during natural activity different initial high-frequency trains of action potentials may lead to faster and stronger contractions.

In conclusion, the doublet at the beginning of motoneuronal activity can be observed in various mammals, and should be considered as a universal mechanism that enables rapid enhancement of force developed by muscles at the beginning of their activity, which lasts despite the passing after-doublet decrease of motoneuronal discharge frequency. Apart from this observation, the influence of doublets on further discharges in motoneuronal firing pattern, especially during voluntary movements, remains unclear.

## Conflict of Interest Statement

The authors declare that the research was conducted in the absence of any commercial or financial relationships that could be construed as a potential conflict of interest.
